# Functions of Nitric Oxide (NO) in Roots during Development and under Adverse Stress Conditions

**DOI:** 10.3390/plants4020240

**Published:** 2015-05-22

**Authors:** Francisco J. Corpas, Juan B. Barroso

**Affiliations:** 1 Group of Antioxidants, Free Radicals and Nitric Oxide in Biotechnology, Food and Agriculture, Department of Biochemistry, Cell and Molecular Biology of Plants, Estación Experimental del Zaidín, CSIC, Apartado 419, E-18080 Granada, Spain; 2 Group of Biochemistry and Cell Signaling in Nitric Oxide, Department of Biochemistry and Molecular Biology, University of Jaén, Campus “Las Lagunillas”, E-23071 Jaén, Spain; E-Mail: jbarroso@ujaen.es; 3 Department of Experimental Biology, Center for Advanced Studies in Olive Grove and Olive Oils, University of Jaén, E-23071 Jaén, Spain

**Keywords:** abiotic stress, nitric oxide, reactive nitrogen species, roots, root development

## Abstract

The free radical molecule, nitric oxide (NO), is present in the principal organs of plants, where it plays an important role in a wide range of physiological functions. Root growth and development are highly regulated by both internal and external factors such as nutrient availability, hormones, pattern formation, cell polarity and cell cycle control. The presence of NO in roots has opened up new areas of research on the role of NO, including root architecture, nutrient acquisition, microorganism interactions and the response mechanisms to adverse environmental conditions, among others. Additionally, the exogenous application of NO throughout the roots has the potential to counteract specific damages caused by certain stresses. This review aims to provide an up-to-date perspective on NO functions in the roots of higher plants.

## 1. Introduction

The free radical nitric oxide, which has been demonstrated to be involved in more plant functions than previously thought, has transformed our understanding of plant physiology. Depending on its rate of production, nitric oxide has the dual role of functioning as a signal molecule at low concentrations and as a stress molecule at high concentrations. The latter could be associated with damages to macromolecules caused by processes such as protein nitration. 

Many NO roles are attributable to the family of NO-related molecules known as reactive nitrogen species (RNS). These molecules include peroxynitrite (ONOO^−^), resulting from the reaction of NO with superoxide radical (O_2_^•−^) [1], and *S*-nitrosothiols (SNOs), produced by the reaction of NO with thiol groups [2]. With respect to the latter group, it is particularly worth highlighting the interaction of the tripeptide glutathione (GSH) with NO leading to the formation of *S*-nitrosoglutathione (GSNO) [3,4]. Another important factor to consider in the NO metabolism is the way in which this molecule is endogenously generated in plant cells. At present, there are two main enzymatic pathways based on an L-arginine-dependent nitric oxide synthase and nitrate reductase using nitrite/nitrate as a precursor [5]. However, other alternative sources such as the pool of *S*-nitrosothiols cannot be ruled out.

## 2. Nitric Oxide Function in Root Architecture

The root architecture system consists of the coordinated growth of primary, lateral and adventitious roots in a process that can be regulated by multiple genetic and environmental factors. The development of the root system can be crucial in determining the survival of the whole plant, especially under adverse environmental conditions [6,7], and consequently in restricting plant productivity in agronomical terms [8]. Although root architecture uses several secondary messengers, including calcium and reactive oxygen species (ROS), nitric oxide (NO) has increasingly come to be regarded as a novel signal molecule in the past decade.

**Figure 1:**
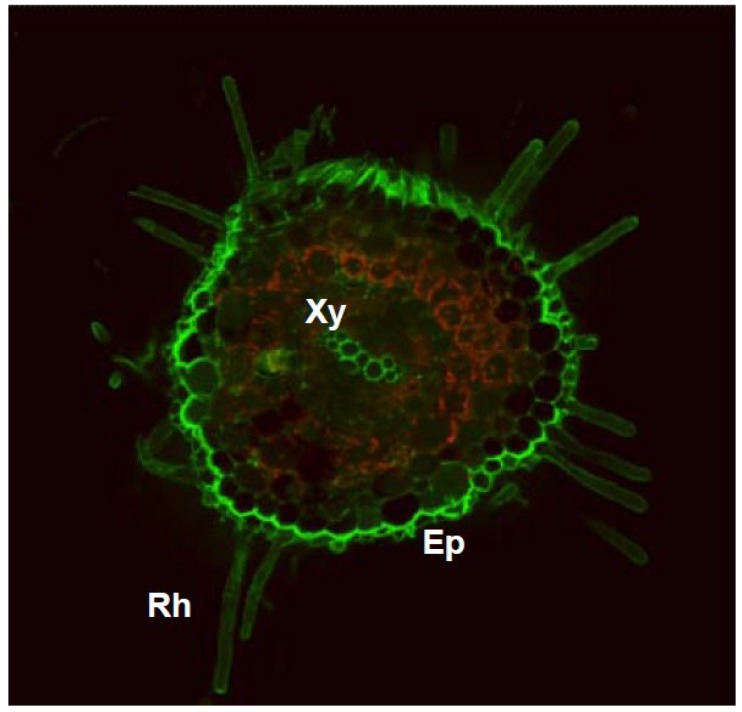
A representative image illustrating the CLSM detection of endogenous NO (green color) in a cross section of a pepper root using 10 mM DAF-FM DA as a fluorescent probe. The orange-yellow color corresponds to the autofluorescence. Ep, epidermis; Rh, root hair; Xy, xylem. Reproduced, with permission, from [16] (Japanese Society of Plant Physiologists, JSPP).

Biochemical and cellular analyses have demonstrated the presence of NO and NO-derived molecules in roots, thus highlighting their importance during root development [9,10,11,12,13,14,15]. Using confocal laser scanning microscopy (CLSM) and specific NO-sensitive fluorophores, such as 4,5-diamino-fluorescein diacetate (DAF-2 DA), analysis of cross sections of pea primary roots revealed a high rate of NO accumulation in epidermal and vascular tissues (xylem), while less intense rates of NO have also been detected in some cells in the cortex [11]. A temporal correlation between root development and NO production from l-arginine-dependent nitric oxide synthase activity has also been observed [11]. Similarly, the presence of other derived molecules, such as *S*-nitrosoglutathione and peroxynitrite, has also been reported in the roots of several plant species, including pepper, pea and *Arabidopsis* [16,17,18,19], indicating that roots have an active NO metabolism. Figure 1 provides a representative cross section showing cellular NO localization in roots of pepper seedlings.

**Figure 2:**
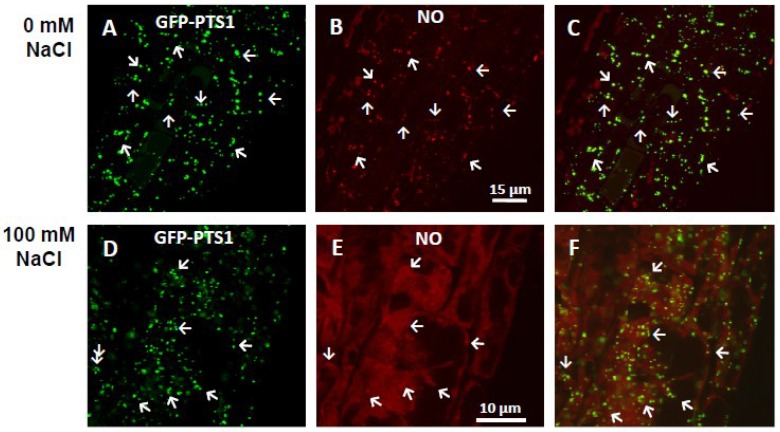
*In vivo* detection of NO (red color) in root peroxisomes of *Arabidopsis* seedlings expressing green fluorescent protein (GFP) through the addition of peroxisomal targeting signal 1 (PTS1) (GFP-PTS1, green color) exposed to 100 mM NaCl. (**A**,**D**) Fluorescence punctuates (green) attributable to GFP-PTS1 indicating the localization of peroxisomes (white arrows) in *Arabidopsis* roots; (**B**,**E**) Fluorescence punctuates (red) attributable to NO detection in the same root area of panel A and D, respectively. (**C**,**F**) Merged image of corresponding panels showing colocalized fluorescence punctuates (yellow). Nitric oxide was detected with diaminorhodamine-4M acetoxymethyl ester (DAR-4M, excitation 543 nm; emission 575 nm) and peroxisomes with green fluorescence protein through the addition of peroxisomal targeting signal 1 (GFP-PTS1, (excitation 495 nm; emission 515 nm). White arrows indicate the localization of peroxisomes. Reproduced, with permission, from [28] (American Society of Plant Biologists, ASPB).

Through the use of exogenous NO donors, NO has also been shown to participate in the induction of root tip elongation [20] and the formation of lateral and adventitious roots [21,22]. The exogenous NO appears to affect the expression of cell cycle regulatory genes and to modulate cellulose synthesis [22,23,24] as well as lignin composition [25]. In addition, the application of exogenous NO could mediate auxin responses during the adventitious rooting process in cucumber seedlings [21]. Recently, analysis of the function of two auxins, indole-3-acetic acid (IAA) and indole-3-butyric acid (IBA), in lateral root formation has highlighted the involvement of peroxisomes. This is explained by the fact that the peroxisomal conversion of IBA to IAA leads to the concomitant generation of NO in these organelles, thus indicating that peroxisomes are dynamically involved in auxin-induced root organogenesis [26]. Figure 2 (panels A to C) shows *in vivo* NO localization in the root tip of *Arabidopsis thaliana*.

During root development, modulations of the content of NO and some related molecules have been observed. Accordingly, a comparative analysis of NO, ONOO^−^ and protein nitration in roots of young and senescent pea plants reveals a general increase in these molecules accompanied by a rise in the antioxidative enzyme (superoxide dismutase and catalase) activity when plants age [18]. As an increase in protein nitration could be regarded as a marker of nitrosative stress [19], it has been suggested that the roots undergo nitrosative stress during senescence. Using proteomic techniques, this study has identified a total of 16 nitrotyrosine-immunopositive proteins, including endochitinase, alcohol dehydrogenase, fructose-bisphosphate aldolase, peroxidase and NADP-isocitrate dehydrogenase (NADP-ICDH). The latter enzyme catalyzes the oxidative decarboxylation of l-isocitrate to 2-oxoglutarate leading to the production of the reduced coenzyme NADPH [27], which is involved in the carbon and nitrogen metabolism, redox regulation and responses to oxidative stress. A comparative analysis of NADP-ICDH activity between young and senescent pea roots shows that this activity is down-regulated in senescent roots, with a more in-depth molecular analysis revealing that nitration at Tyr392 of NADP-ICDH is responsible for this inhibition [18].

Hemin is an iron-containing porphyrin present in a variety of proteins and capable of inducing heme oxygenase-1, which catalyzes the initial and rate-limiting step of the oxidative degradation of heme and generates biliverdin, free iron (Fe^2+^) and carbon monoxide (CO). Using cucumber (*Cucumis sativus*) seedlings, the exogenous application of hemin has been shown to induce heme oxygenase-1 activity, with a concomitant NO production, and also formation of adventitious roots [29]. These authors also demonstrate that this response is blocked by various NOS-like activity inhibitors.

As part of a study of the root’s ability to acquire mineral from soil, different sets of experiments have verified that NO can, for example, modulate iron acquisition by roots. Thus, NO combined with ethylene is not only capable of inducing the expression of Fe acquisition genes [30], but can also act downstream of auxin to trigger ferric-chelate reductase (FCR) activity at the plasma membrane in order to enhance Fe uptake [31].

## 3. Involvement of NO in the Interaction of Roots with Beneficial Microorganisms: Nodules and Mycorrhiza

Some major bacterial and fungal groups of microorganisms can establish beneficial interactions with plants through their roots, with the two most studied being Rhizobium-legume and arbuscular mycorrhizal fungi-legume [32,33].

With respect to functional root nodules, which enable nitrogen gas to be converted into ammonia, there is a complex signaling cascade between the legume and rhizobia [34], with NO being involved in the establishment and functioning of these interactions [35,36,37,38,39,40]. In the root nodule, two essential elements, leghemoglobin (Lb) and nitrogenase, are present in order to ensure efficient nitrogen-fixing. Lb is an oxygen carrier whose function is to prevent the presence of O_2_, which inactivates nitrogenase, the enzyme responsible for fixing atmospheric nitrogen to ammonia. This activity is inhibited by NO, indicating that NO levels in rhizobia are a determining factor in efficient symbiosis processes [40]. Thus, in senescing nodules, an increase in ROS and RNS has been reported to cause nitro-oxidative stress, leading to a reduction in the ability of symbiotic leghemoglobins to scavenge oxygen due to modifications mediated by these ROS/RNS [41,42]. Recently, the formation of nitrated leghemoglobins during the normal metabolism in functional nodules has also been reported, which may act as a sink for toxic peroxynitrite and consequently play a protective role in the symbiosis [43].

Similarly, root colonization by arbuscular mycorrhizal fungi also requires a whole series of events to occur. Recently, it has been confirmed that NO is produced in the roots of *Medicago truncatula* when they come in contact with the exudates of the fungus *Gigaspora margarita* [44]. In addition, preliminary data indicate that NO is involved in the interaction of olive seedling roots with the arbuscular mycorrhizal fungus *Rhizophagus irregularis* [45].

## 4. Involvement of NO in Root System under Environmentally Adverse Conditions

Plant root systems are directly exposed to a wide range of environmentally adverse conditions that affect the nutrition status and/or integrity of the root system and consequently the survival of whole plants. NO has been studied in relation to adverse conditions, including drought, flooding, mineral deficiency, salinity, heavy metal and pathogens [46,47,48,49,50,51]. Many of these situations are accompanied by stress conditions that usually have important nitro-oxidative stress components [19]. As previously mentioned, NO can act as a signal molecule or as part of a mechanism producing a local and/or systemic response. Therefore, NO production in roots under specific adverse conditions can differ depending on factors such as the age of plants, stress intensity and exposition time.

Cadmium (Cd^2+^) is a non-essential toxic heavy metal, which has a negative impact on plant growth [52]. Accumulated data now show that Cd^2+^ induces nitro-oxidative stress, which affects the root system and, especially, NO homeostasis [53]. In 28-day-old pea (*Pisum sativum)* plants exposed to 50 µM CdCl_2_ for 14 d, a significant reduction in root growth was observed, mainly in relation to the number and length of lateral roots. These morphological changes are accompanied by a decrease in NO content in roots [54]. However, responses can differ depending on plant species, age and time of exposition to Cd^2+^. Thus, in three-day-old yellow lupine (*Lupinus luteus* L.) seedling roots exposed to 89 μM CdCl_2_, programmed cell death preceded by a NO burst in the root tips occurred [55]. In the roots of three-week-old *Arabidopsis thaliana* seedlings grown on Petri plates and then treated with 200 μM CdCl_2_ for 7 h, an induction of NO generation was clearly observed [56]. In another study, 14-day-old *A. thaliana* transgenic seedlings expressing cyan fluorescent protein, through the addition of peroxisomal targeting signal 1 (PTS1), were used to visualize peroxisomes *in vivo* exposed to 150 µM CdCl_2_. Under these conditions, an intensification of NO production in root tips, specifically in peroxisomes, accompanied by a concomitant increase in the superoxide radical and peroxynitrite involved in the generation of nitro-oxidative stress, was reported [57].

On the other hand, heavy metals, such as zinc (Zn^2+^), an essential micronutrient naturally present in soils, can be accumulated and consequently also induce oxidative stress [58]. In Brassica, 300 µM Zn^2+^ triggers changes in root architecture and the cell wall structure. Moreover, these modifications are accompanied by a significant overproduction of NO, ONOO^−^ and an accumulation of nitrated proteins [59].

Arsenic (As), a toxic metalloid for plants, can be incorporated as arsenite (As III) throughout the aquaporin channels and as arsenate (AsV) by the phosphate transporter system. Arsenic adversely affects photosynthesis, respiration, growth regulation and reproduction. In seven-day-old *A. thaliana* seedlings, grown on Petri plates and then treated with 500 μM KH_2_AsO_4_, corresponding to As(V) for an additional seven days, a significant increase in the NO content in roots has been observed. This was also accompanied by an increase in tyrosine nitration as well as a concomitant reduction in the GSH and GSNO content [60].

Plant roots are usually prone to halotropism, as root growth tends to occur away from highly saline environments [61]. Halotropism can affect intracellular ion homeostasis, the primary carbon metabolism, plant growth and development through ion toxicity, induced nutritional deficiency, water deficits and oxidative stress [62]. In *A. thaliana* seedlings grown under salinity conditions (100 mM NaCl), a significant increase in NO accompanied by an increase in the superoxide radical and peroxynitrite was observed in root tips, which leads to a nitro-oxidative stress [63]. As with cadmium stress, root peroxisomes also appear to be actively involved in NO generation under salinity stress conditions (Figure 2) [28]. With respect to this NO signaling mechanism in salinity stress situations, both osmotic stress-activated protein kinase and glyceraldehyde-3-phosphate dehydrogenase appear to form a cellular complex and to be directly or indirectly regulated by NO [64]. In this context, Liu *et al*. [65] have shown that 100 mM NaCl stress reduces *Arabidopsis* root meristem size by increasing NO accumulation, which represses the expression of *PINFORMED (PIN) genes.* In consequence, the auxin levels are reduced and also the auxin signaling [65] because these *PIN* genes encode for transmembrane proteins involved in the transport of auxin [66].These data are in good agreement with previous observations which indicated the interplay between NO and auxin [67,68,69]. 

## 5. Nitric Oxide and Its Biotechnological Applications 

With respect to the potential biotechnological applications of NO in higher plants, there is an increasing amount of data showing that pre-treatments of plant roots with different types of NO donors can stimulate response mechanisms. This not only prevents nitro-oxidative damages in the roots themselves but also protects the aerial part of the plants against certain abiotic stress situations, thus highlighting the signaling function of NO.

For example, in *Lupinus luteus*, exogenous NO applications counteract the inhibitory effect of heavy metals and salinity on root growth [20]. In some cases, such as that of maize seedlings, this beneficial effect of NO has been shown to be caused by an increase in Na^+^/H^+^ antiporter activity in the tonoplast [70]. However, in other cases, increased resistance induced by exogenous NO is due to stimulation from the antioxidant system. For example, the application of 50 µM sodium nitroprussiade (SNP) as NO donor stimulates ROS-scavenging enzymes and reduces the accumulation of H_2_O_2_ in the mitochondria of cucumber (*Cucumis sativus* L.) roots induced by 100 mM NaCl [71]. In another case, pre-treatment of *Citrus aurantium* L. plant roots with 100 µM SNP for 48 h before treatment with 150 mM NaCl results in a considerable reduction in visible injury and alleviates the physiological effects of salinity stress [72]. Similarly, in tomato (*Solanum lycopersicum*) plants, the addition of 300 µM SNP to roots has been shown to significantly increase resistance to NaCl toxicity, which is reflected in the growth and chlorophyll content of plants exposed to NaCl. Moreover, exogenous SNP also decreases NaCl-induced lipid oxidation levels in leaves and induces an increase in the activities of the antioxidant system including superoxide dismutase, ascorbate peroxidase, glutathione reductase and peroxidases in roots and leaves as well as the content of ascorbate and proline [73].

As low levels of Zn availability affect crop yield and food production worldwide. The application of NO through the addition of 100 μM GSNO has been shown to modulate Zn acquisition in wheat plants grown at supra-optimum, non-toxic Zn concentrations [74].

Another example concerns arsenic toxicity, with the application of NO (50 μM SNP) creating resistance to As in *Oryza sativa*. This reduces malondialdehyde, superoxide radical and H_2_O_2_ content and also increases antioxidant activities of enzymes, such as superoxide dismutase, ascorbate peroxidase, guaiacol peroxidase and catalase [75]. Similar results have been described in roots of wheat and alfalfa under aluminum-induced oxidative stress [76,77].

In summary, available data suggest that the exogenous application of NO throughout the plant roots could ameliorate nitro-oxidative stress induced in plants. Future research in this area under field conditions would make a positive contribution to developing sustainable crops.

## 6. Conclusions and Perspectives

There is currently no doubt that nitric oxide combined with other molecules is an important component in the functioning and physiology of plant roots. Thus, NO is involved in root architecture, nutrient acquisition and microorganism interactions and also in the mechanism of response to adverse environmental conditions, among others. Moreover, the exogenous application of NO throughout the roots has the potential to counteract specific damage caused by certain stresses, which should open up the possibility of using NO to develop new biotechnological applications. Although significant advances in our understanding of the main role played by NO in plant roots have been made, many gaps remain in our knowledge of the specific targets that determine its cellular functions.
